# Ubiquitin-specific protease 13 promotes colorectal cancer progression by stabilizing mitogen-activated protein kinase kinase 3

**DOI:** 10.1186/s43556-025-00375-3

**Published:** 2025-11-27

**Authors:** Si-yu Chang, Bo Gu, Yong Xiong, Meng-si Zhao, Le Li, Yi Liu, Bai-qi Wang, Guo-qing Li, Run-lei Du, Xiao-dong Zhang

**Affiliations:** 1 National Health Commission Key Laboratory of Birth Defect Research and Prevention, MOE Key Lab of Rare Pediatric Diseases, Hunan Provincial Key Laboratory of Basic and Clinical Pharmacological Research of Gastrointestinal Cancer, The Second Affiliated Hospital, School of Basic Medical Sciences, Hengyang Medical School, University of South China, Hengyang, China; 2 Hubei Key Laboratory of Cell Homeostasis, College of Life Sciences, Wuhan University, Wuhan, Hubei 430072 China

**Keywords:** Colorectal cancer, USP13, MKK3, P38 MAPK signaling, Deubiquitination, Tumor progression

## Abstract

The deubiquitinase ubiquitin-specific protease 13 (USP13) has been implicated in various cancers, yet its precise molecular function and clinical significance in colorectal cancer (CRC) remain poorly defined. Here, we identify USP13 as a critical regulator of CRC progression through systematic investigation of its impact on oncogenic signaling pathways. Using luciferase-based pathway screening, we discovered that USP13 activates the mitogen-activated protein kinase (MAPK) signaling cascade. USP13 directly interacts with and stabilizes mitogen-activated protein kinase kinase 3 (MKK3), a key upstream kinase of the p38/MAPK pathway, through removal of K48-linked ubiquitination at the K32 residue. This deubiquitination process requires the UBA domain of USP13 and prevents proteasomal degradation of MKK3, leading to enhanced p38 phosphorylation and activation. Functional validation demonstrated that USP13 and MKK3 significantly promotes CRC cell proliferation, migration, and invasion in vitro. Importantly, in vivo xenograft experiments confirmed that USP13-driven tumor growth depends on MKK3 and can be rescued by constitutive p38 activation. Clinical correlation analysis of CRC patient specimens revealed a strong positive correlation between USP13 and MKK3 expression levels, with elevated USP13 expression associated with advanced disease stage. Our findings not only establish the USP13-MKK3-p38 axis as a crucial molecular pathway in CRC progression but also identify USP13 as a promising therapeutic target.

**Supplementary Information:**

The online version contains supplementary material available at 10.1186/s43556-025-00375-3.

## Introduction

Colorectal cancer (CRC) is the third most commonly diagnosed malignant tumor and the second leading cause of cancer-related deaths worldwide [1]. Aberrant signaling pathways, such as Wnt/β-catenin, RAS/RAF/MEK/ERK, PI3K/AKT, p53, TNFα and TGF-β, lie at the core of CRC pathogenesis by driving tumorigenesis, cell proliferation, and metastasis [2]. Targeted therapy against these key pathways represents a promising strategy for CRC treatment [3–5].

The mitogen-activated protein kinase (MAPK) is a key driver of CRC, where its dysregulation directly fuels tumor progression. Frequent mutations in critical components not only serve as essential diagnostic biomarkers but also establish this pathway as a pivotal target for therapeutic intervention [6, 7]. The MAPK family member p38 kinases are primarily activated by phosphorylation kinases such as MKK3 (mitogen-activated protein kinase kinase 3) and MKK6 [8, 9]. Once activated, p38 phosphorylates a wide range of substrates, including Elk-1, c-Jun, ATF2, p53, and c-MYC [10]. While p38 exhibits a dual function in various cancers [11], MKK3 also plays a similarly paradoxical role, capable of both suppressing and promoting tumorigenesis, including breast cancer, liver cancer, cervical and ovarian cancer, and lung cancer [12]. In CRC, gain-of-function p53 mutants transcriptionally upregulates MKK3 and promotes cell proliferation [13]. MKK3 binds to the oncoprotein c-Myc and enhances its stability and transcriptional activity, implying MKK3 may function as a more extensive signaling hub than previously recognized [14]. Increased MKK3 level correlates with poor prognosis in CRC patients and emerges as a promising therapeutic target [15–17].

Ubiquitination is a crucial post-translational modification (PTM) that regulates protein stability and cellular signaling transduction [18]. This process is tightly controlled by multiple E3 ligases and can be reversed by deubiquitinases (DUBs) [19]. Previous studies have shown that FBXO31 (F-Box only protein 31) mediates K48-linked polyubiquitination and degradation of MKK6 [20], and TRIM9s (Tripartite Motif protein 9 short isoform) promotes K63-linked ubiquitination of MKK6, which in turn prevents its degradation [21]. Despite these findings, the regulatory landscape of E3 ligases and DUBs that specifically target p38/MAPK or its upstream kinases MKK3/6 remains largely unexplored [22]. This regulatory gap underscores the critical importance of exploring ubiquitination modifications of MKK3.

USP13 (Ubiquitin Specific Peptidase 13) is a multifunctional deubiquitinating enzyme involved in various cellular processes, including DNA damage repair [23, 24], autophagy [25, 26], ferroptosis [27], inflammation [28] and immune regulation [29, 30]. USP13 expression is driven by oncogenic KRAS^G12C^ in non-small cell lung cancer (NSCLC) through Ras-responsive element-binding protein 1 (RREB1). Overexpressed USP13 removes K63-linked polyubiquitination of β-catenin, promoting KRAS^G12C^ NSCLC metastasis [31]. While the functions of USP13 have been extensively studied in various cancers, including gastric cancer [32], melanoma [33], ovarian cancer [34], cervical cancer [35], cholangiocarcinoma [36], glioblastoma [37], kidney cancer [38], and breast cancer [39, 40], its role in CRC remains unclear. Given its involvement in key cellular processes, USP13 represents a promising therapeutic target for cancer treatment, antiviral defense, and metabolic disorders [41].

This study identifies USP13 as the first DUB for MKK3, which stabilizes MKK3 and thereby potentiates p38/MAPK signaling in CRC cells. Mechanically, USP13 removes K48-linked polyubiquitination of MKK3 at Lys32 residue and preventing MKK3 from proteasomal degradation. Genetically deletion of USP13 or MKK3 suppressed CRC cell proliferation, migration in vitro and tumor formation in vivo, effects that were synergistically enhanced by the pharmacological inhibition of p38 signaling. Therefore, this study delineates a previously unrecognized ubiquitin-dependent regulatory mechanism for MKK3-p38/MAPK signaling and highlights the considerable therapeutic potential of combinatorial targeting of this pathway in CRC.

## Results

### USP13 promotes colorectal cancer progression

**Fig. 1:**
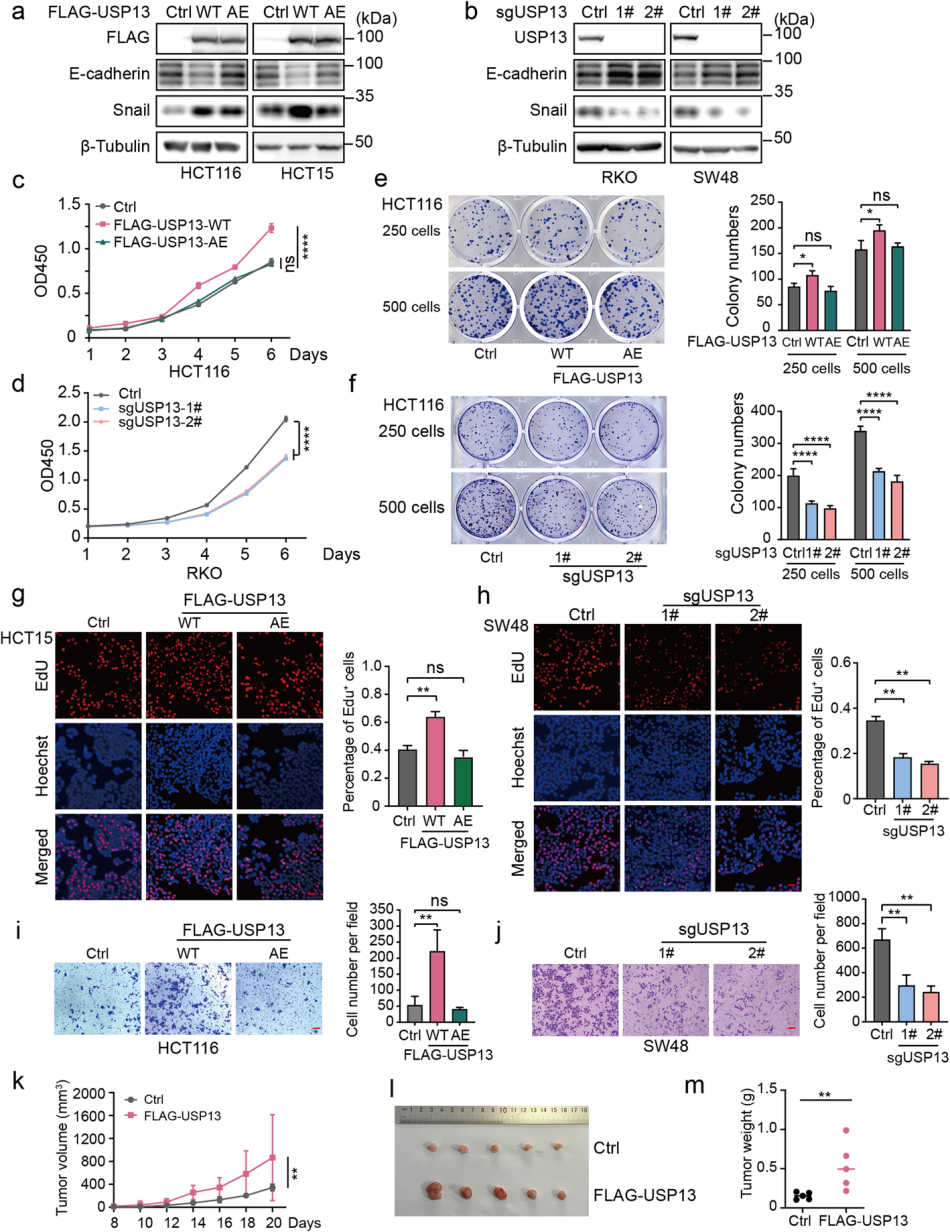
USP13 promotes colorectal cancer progression. **a** Western blot was performed to analyze the expression of FLAG-USP13 in HCT116 and HCT15. **b** Western blot was performed to detect the expression of USP13 in CRC cells infected with sgUSP13 lentivirus. **c** Cell proliferation was assessed by CCK8 assay in FLAG-USP13 or FLAG-USP13-AE HCT116 cells. **d** Cell proliferation was assessed by CCK8 assay in sgUSP13 RKO cells. **e**–**f** Colony formation ability was determined in HCT116 cells. **g**-**h** EdU assay was performed to determine the DNA synthesis activity in CRC cells. Representative images were presented. Scale bar, 100 μm. **i**-**j** Transwell assay was performed to determine the migration ability of RKO cells. Representative images were presented. Scale bar, 100 μm. **k-m** HCT116 cells were subcutaneously injected into the nude mice (*n* = 5 per group). The tumor growth curve (**k**), photographs (**l**) and weight (**m**) were represented. The date were presented as means ± SD at least three independent experiments. Student’s *t* -tests (**e**-**j** and **m**) and Two-way ANOVA test (**c**, **d** and **k**) were performed with mean ± SD, ns: no significance, *p < 0.05, **p < 0.01, ***p < 0.001, ****p < 0.0001

To investigate the clinical relevance of USP13 in CRC, we analyzed transcriptomic data from public databases and found that USP13 was significantly upregulated in colon adenocarcinoma (COAD) from the GEPIA2 (Gene Expression Profiling Interactive Analysis) (Fig. S1). To determine whether USP13 contributes to malignant phenotypes, we established HCT116 and HCT15 cell lines stably overexpressing wild-type USP13 (USP13-WT) or its catalytically inactive mutant USP13-AE (C345A/M664/739E) (Fig. 1a), and generated USP13-knockout RKO and SW48 cells via CRISPR/Cas9 (Fig. 1b). Knockdown of USP13 led to a decrease in Snail and an increase in E-cadherin, while overexpression had the opposite effect. CCK8 assays demonstrated accelerated growth rates in USP13-overexpressing HCT116 cells, while USP13-AE-expressing cells showed no significant difference (Fig. 1c). Conversely, USP13-knockout HCT116 cells exhibited growth retardation (Fig. 1d). USP13 overexpression promoted colony formation, whereas the USP13-AE mutant had no such effect (Fig. 1e-f). In HCT15 cells, USP13 overexpression increased DNA synthesis, but the USP13-AE mutant did not (Fig. 1g). Conversely, USP13 knockout reduced DNA synthesis in SW48 cells (Fig. 1h). In HCT116 cells, USP13 overexpression enhanced migration, but the USP13-AE mutant did not (Fig. 1i). By contrast, migration was impaired in USP13 knockout cells (Fig. 1j). In vivo xenograft experiments demonstrated that USP13-overexpressing HCT116 cells formed larger tumors with heavier weight compared to controls (Fig. 1k-m). These findings demonstrate that USP13 is frequently upregulated in CRC and promotes multiple malignant phenotypes in a deubiquitinase activity-dependent manner.

### USP13 interact with MKK3

**Fig. 2:**
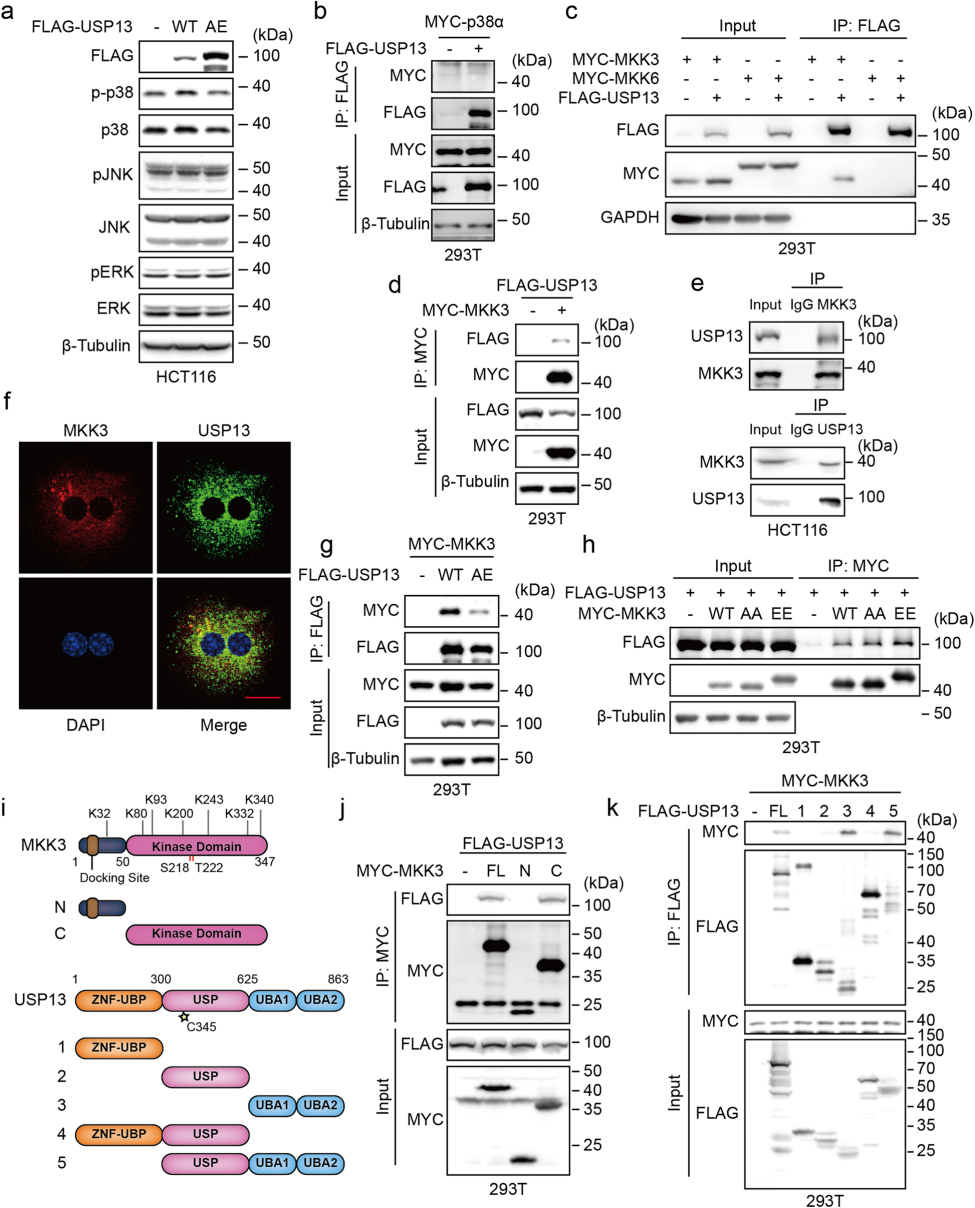
USP13 interacts with MKK3. **a** HCT116 cells overexpressing FLAG-USP13 or FLAG-USP13-AE were harvested for Western blot with the indicated antibodies. **b** Lysates of HEK293T cells transfected with MYC-p38α and FLAG-USP13 as indicated were immunoprecipitated with anti-FLAG beads, followed by Western blot with the indicated antibodies. **c** Lysates of HEK293T cells transfected with MYC-MKK3, MYC-MKK6 and FLAG-USP13 as indicated were immunoprecipitated with anti-FLAG beads, followed by Western blot with the indicated antibodies. **d** Lysates of HEK293T cells transfected with MYC-MKK3, FLAG-USP13 as indicated were immunoprecipitated with anti-MYC beads, followed by Western blot with the indicated antibodies. **e** Immunoprecipitation were performed using MKK3 or USP13 antibodies, followed by Western blot to identify the interaction between endogenous USP13 and MKK3. **f **The colocalization of USP13 and MKK3 was detected with immunofluorescence. MKK3 was labeled with red fluorescence, USP13 with green fluorescence, and the nucleus was stained with DAPI. Scale bar, 5 μm. **g** Lysates of HEK293T cells transfected with MYC-MKK3, FLAG-USP13-WT or AE were immunoprecipitated with anti-FLAG beads, followed by Western blot with the indicated antibodies. **h** Lysates of HEK293T cells transfected with empty vector, MYC-MKK3, MYC-MKK3-AA or EE, together with FLAG-USP13, were immunoprecipitated with anti-MYC antibody, followed by Western blot. **i** Schematic diagram of MKK3 and USP13 and their truncation mutants. **j** MYC-MKK3 truncations and FLAG-USP13 were transfected into HEK293T cells, followed by co-immunoprecipitation with anti-MYC antibody. **k** FLAG-USP13 truncations and MYC-MKK3 were transfected into HEK293T cells, followed by co-immunoprecipitation with anti-FLAG antibody

To identify the signaling pathways involved in USP13-mediated promotion of CRC progression, we conducted a preliminary screen with a luciferase reporter assay and found that USP13 overexpression enhanced the activation of the MAPK signaling pathway (Fig. S2). Western blot analysis revealed that USP13 overexpression selectively increased p38 phosphorylation, without altering total p38 or other MAPK protein levels (Fig. 2a). Although USP13 did not directly bind p38 (Fig. 2b), it specifically interacted with the upstream kinase MKK3, as confirmed by co-immunoprecipitation (Co-IP) and endogenous Co-IP assays (Fig. 2c–e). Immunofluorescence further showed cytoplasmic co-localization of USP13 and MKK3 (Fig. 2f). Furthermore, The interaction was dependent on USP13 catalytic activity, as the USP13-AE mutant displayed reduced binding (Fig. 2g). Intriguingly, enhanced binding of USP13 was observed with the constitutively active MKK3-EE mutant compared to its kinase-dead form (MKK3-AA), indicating that the interaction is phosphorylation-dependent (Fig. 2h). Subsequent domain mapping identified the UBA domain of USP13 and the C-terminal region of MKK3 as essential for the interaction (Fig. 2i–k).

In summary, USP13 selectively activates p38 phosphorylation by binding to MKK3 in a catalytically and phosphorylation dependent manner, indicating a functional USP13–MKK3–p38 axis in CRC.

### USP13 promotes the stabilization of MKK3

**Fig. 3:**
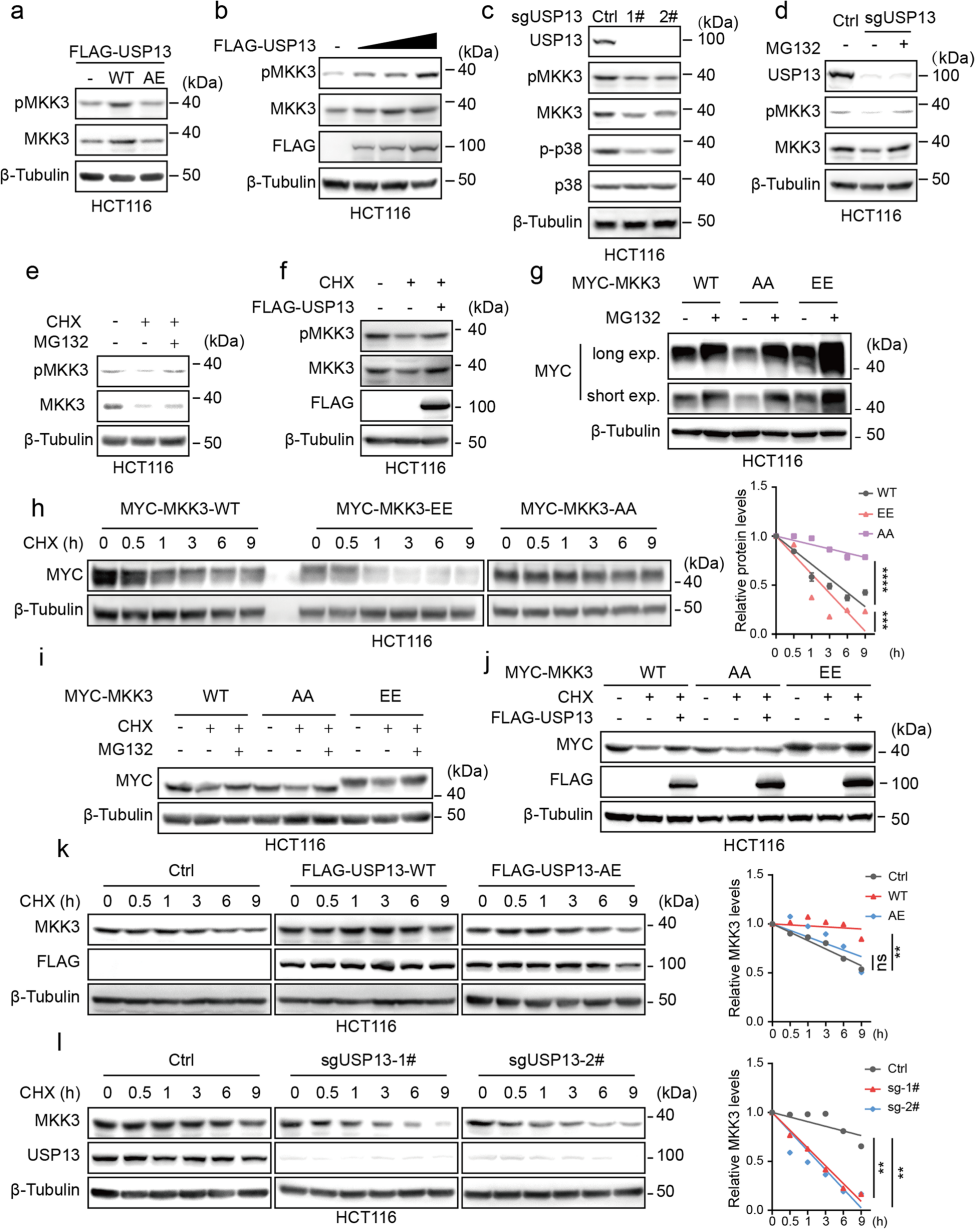
USP13 promotes the stabilization of MKK3. **a** Western blot was performed to detect the protein levels of MKK3 and pMKK3 in HCT116 cells overexpressing FLAG-USP13-WT or AE. **b** A gradient transfection of FLAG-USP13 was performed, followed by Western blot with the indicated antibodies. **c** Western blot was performed to detect the protein levels of MKK3, pMKK3, p38, and p-p38 in control and USP13 knockout HCT116 cells. **d** Control and USP13 knockout HCT116 cells untreated or treated with MG132 (10 μM,12 h) were subjected to Western blot with the indicated antibodies. **e** CHX (50 μg/mL) and MG132 (10 μM) was added to HCT116 cells for 12 h, followed by Western blot with the indicated antibodies. **f** FLAG-USP13 was transfected into HCT116 cells, and 24 h later, CHX (50 μg/mL) was added for 12 h. Western blot was performed using the indicated antibodies. **g** MG132 (10 μM) was added to HCT116 cells stably overexpressing MYC-MKK3-WT or AA/EE for 12 h. The expression levels of the exogenous protein were detected using the MYC antibody. **h** CHX (50 μg/mL) was added to HCT116 cells stably overexpressing MYC-MKK3 or AA/EE, and cells were collected at the indicated time points, followed by Western blot with the indicated antibodies. **i** HCT116 cells expressing MYC-MKK3 or AA/EE were treated with CHX (50 μg/mL) and MG132 (10 μM) for 12 h, and followed by Western blot with the indicated antibodies. **j** HCT116 cells expressing MYC-MKK3 or AA/EE were transfected with FLAG-USP13. After 24 h, CHX (50 μg/mL) was added for 12 h, and followed by Western blot with the indicated antibodies. **k-l** CHX (50 μg/mL) was added to HCT116 cells, and cells were collected at the indicated time points, followed by Western blot with the indicated antibodies. ImageJ was used to quantify the gray values of the proteins at each time point, and relative values were calculated by comparing with the internal control, setting the 0-h time point as the baseline. A line graph was plotted to show the protein levels over time (**h, k, l**). The date were presented as means ± SD at least three independent experiments. Two-way ANOVA test was performed. ns: no significance, **p < 0.01, ***p < 0.001, ****p < 0.0001

We next asked whether USP13, as a deubiquitinating enzyme, stabilizes its binding partner MKK3. As shown in Fig. 3a and b, USP13 overexpression enhanced MKK3 protein levels, whereas its catalytically inactive mutant (AE) failed to do so. Accordingly, the accumulation of MKK3 protein was accompanied by a synchronous increase in its phosphorylation level (Fig. 3a, b,). USP13 knockout in HCT116 cells significantly reduced MKK3 and p-p38 levels (Fig. 3c). In USP13-knockout cells, MG132 treatment restored MKK3 levels (Fig. 3d). CHX treatment significantly reduced MKK3 protein level, which was reversed by MG132 co-treatment (Fig. 3e). Similarly, USP13 overexpression stabilized the MKK3 protein. (Fig. 3f). These results suggest that USP13 may regulate MKK3 protein levels by modulating its proteasomal degradation pathway.

Next, we evaluated the impact of the MKK3 phosphorylation status on its stability. The MKK3-EE mutant exhibited a more pronounced protein level restoration upon MG132 treatment and, moreover, displayed accelerated degradation kinetics in CHX chase assays compared to wild-type and MKK3-AA (Fig. 3g, h). These findings collectively demonstrate that the phospho-mimetic MKK3 is more susceptible to proteasomal degradation. While MG132 co-treatment stabilized all MKK3 variants (Fig. 3i), USP13 specifically rescued MKK3-WT and MKK3-EE but not MKK3-AA (Fig. 3j). Moreover, CHX chase assay demonstrated that overexpression of wild-type USP13, but not the USP13-AE mutant, resulted in an extension of MKK3's half-life and a delay in its degradation (Fig. 3k). Conversely, USP13 knockout accelerated MKK3 degradation (Fig. 3l). In summary, USP13 stabilizes MKK3 by counteracting its proteasomal degradation, in a manner dependent on both the catalytic activity of USP13 and the phosphorylation status of MKK3.

### USP13 targets MKK3 for deubiquitination

**Fig. 4:**
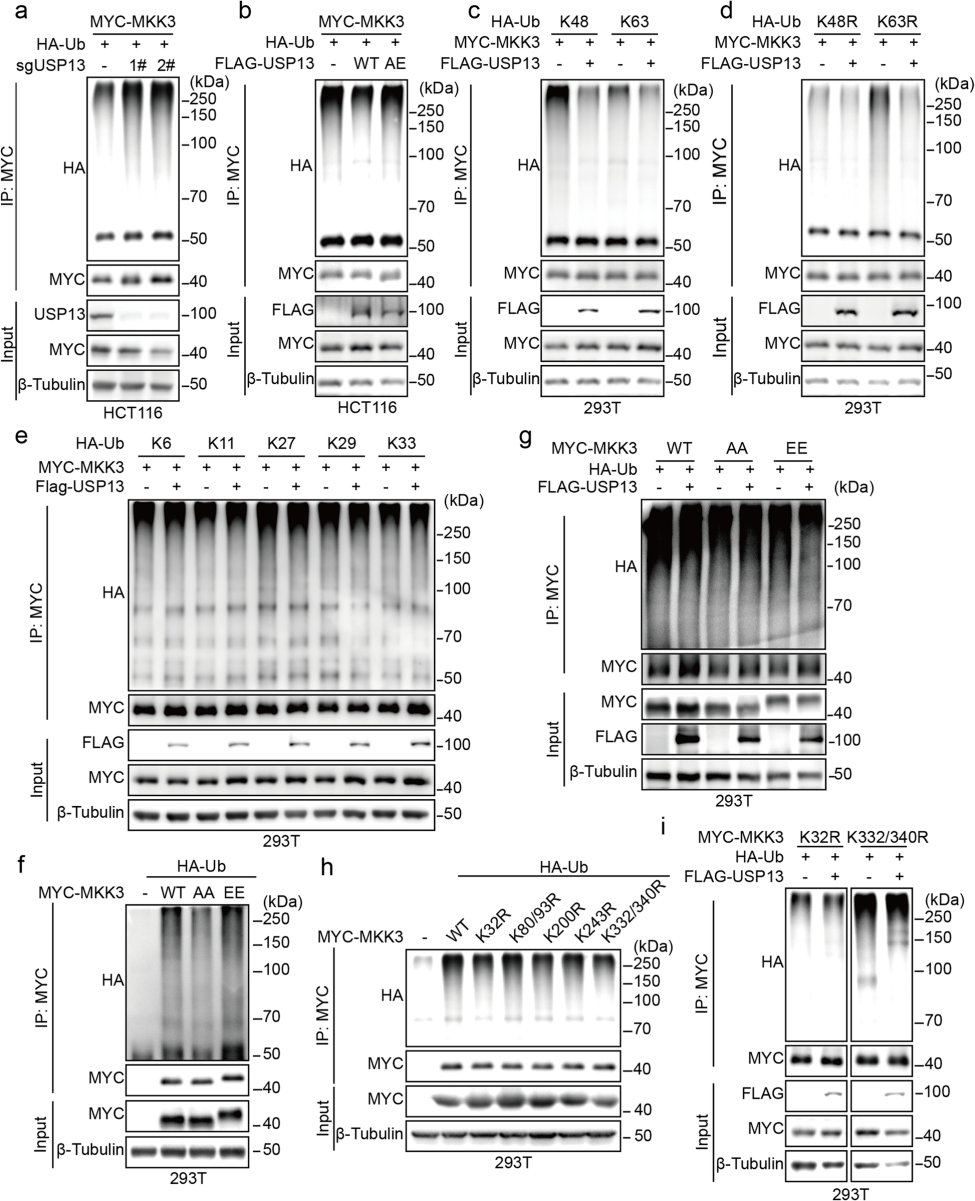
USP13 targets MKK3 for deubiquitination. **a** HCT116 wild-type or USP13 knockout cells were transfected with MYC-MKK3 and HA-Ub, followed by MG132 treatment (10 μM, 12 h). Ubiquitination assay was performed to detect the ubiquitination levels of MKK3. **b** HCT116 cells were transfected with MYC-MKK3 and FLAG-USP13-WT or AE followed by MG132 treatment (10 μM, 12 h). Ubiquitination assay was performed to detect the ubiquitination levels of MKK3. **c-d** HEK293T cells were transfected with FLAG-USP13, MYC-MKK3, and HA-Ub mutants (K48, K63 or K48R, K63R) and treated with MG132 (10 μM, 12 h), followed by ubiquitination assay. **e** Ubiquitination assay was performed to analyze the ubiquitination of MKK3 in HEK293T cells transfected with MYC-MKK3 and HA-Ub mutants (K6, K11, K27, K29 or K33) together with or without Flag-USP13. **f** HEK293T cells were transfected with MYC-MKK3 or its AA/EE mutants as indicated, together with HA-Ub. Cell lysates were subjected to ubiquitination assay. **g** 293 T cells were transfected with MYC-MKK3-WT or AA/EE mutants, HA-Ub, and FLAG-USP13. After 24 h, cells were treated with MG132 (10 μM, 12 h). Cell lysates were subjected to ubiquitination assay. **h** HEK293T cells were transfected with MYC-MKK3 or its mutants (K32R, K80/93R, K200R, K243R, K332/340R) as indicated, together with HA-ubiquitin. Cell lysates were subjected to ubiquitination assay. **i** HEK293T cells were transfected with FLAG-USP13, HA-Ub, and MYC-MKK3 or its mutants (K32R or K332/340R), followed by ubiquitination assay

Based on the finding that USP13 stabilizes MKK3, we investigated its ability to regulate MKK3 ubiquitination. Knockdown of USP13 increased MKK3 ubiquitination (Fig. 4a), and wild-type USP13, but not the catalytically inactive USP13-AE mutant, effectively reduced this modification (Fig. 4b). These results demonstrated that USP13 mediates the deubiquitination of MKK3 in a manner strictly dependent on its enzymatic activity.

To delineate the specific ubiquitin linkage type involved, we employed K48 and K63 ubiquitin mutants. Co-transfection experiments demonstrated USP13 preferentially removes K48-linked polyubiquitin chains from MKK3 (Fig. 4c), a specificity further confirmed through reciprocal mutation analysis (Fig. 4d). Importantly, USP13 exhibited no detectable effect on other ubiquitin linkages, indicating remarkable linkage type selectivity (Fig. 4e).

Intriguingly, the constitutively active MKK3-EE mutant displayed significantly higher basal ubiquitination than its inactive counterpart MKK3-AA (Fig. 4f). This phosphorylation-dependent susceptibility was mirrored in USP13's regulatory preference, as evidenced by significantly reduced ubiquitination of MKK3-EE compared to MKK3-AA upon USP13 overexpression (Fig. 4g).

Guided by these findings, we mapped critical ubiquitination sites through systematic lysine mutagenesis. Among seven candidate residues (K32, K80, K93, K200, K243, K332, K340), the K32R and K332/340 mutations most dramatically reduced basal ubiquitination (Fig. 4h). We identified K32 as the primary site on MKK3 targeted by USP13, as mutation of this residue completely abolished deubiquitination (Fig. 4i).

### MKK3 promotes colorectal cancer progression

**Fig. 5:**
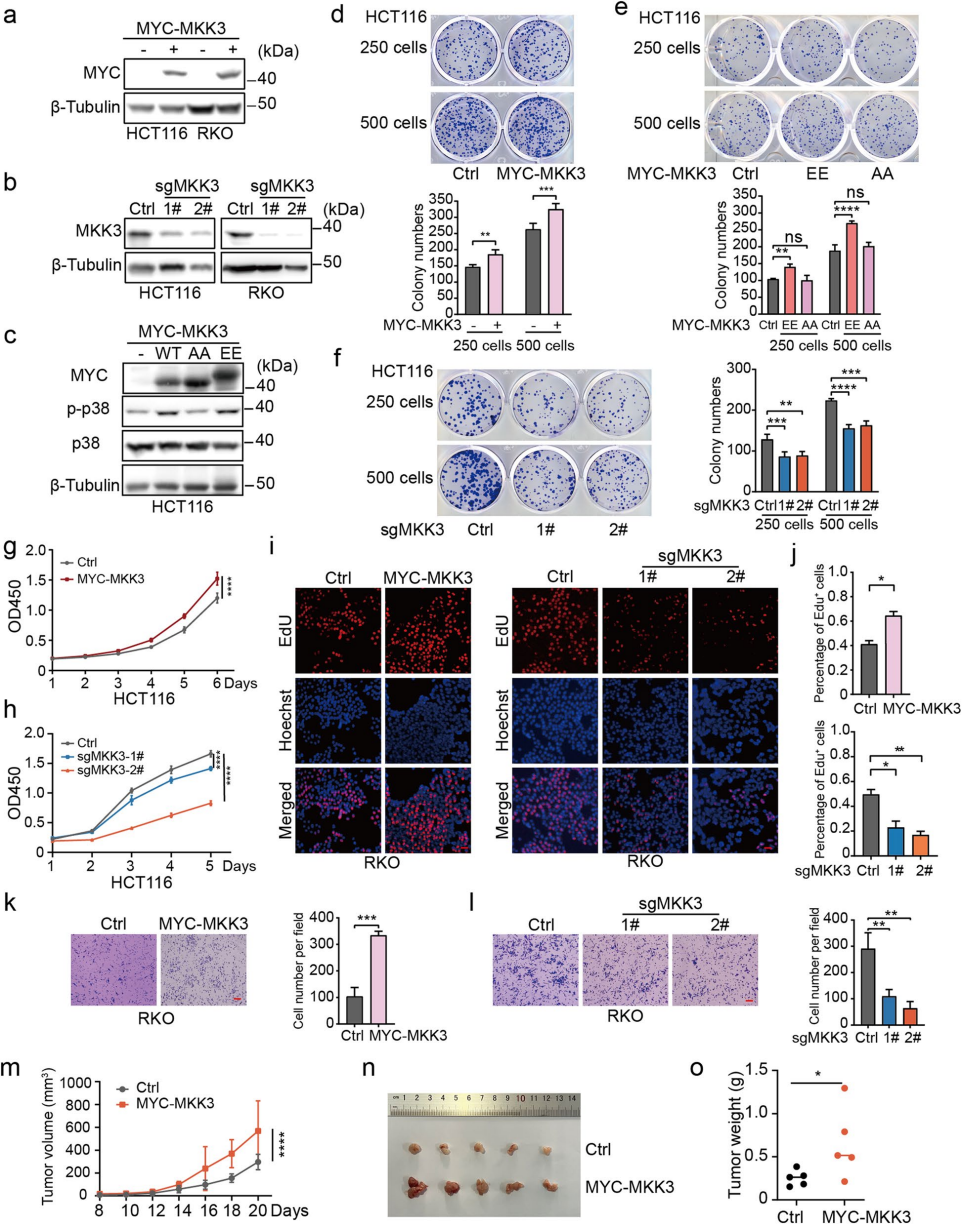
MKK3 promotes colorectal cancer progression. **a** Western blot was performed to detect the expression of MKK3 in CRC cells infected with MYC-MKK3 lentivirus. **b** Western blot was performed to detect the expression of MKK3 in CRC cells infected with sgMKK3 lentivirus. **c** Western blot was performed to detect the expression of MKK3-AA/EE mutants in CRC cells infected with MYC-MKK3-AA/EE lentivirus. **d-f** Colony formation ability was determined in HCT116 cells. **g-h** CCK-8 assay was performed to detect cell proliferation ability of HCT116 cells. **i-j** EdU assay was performed to determine the DNA synthesis activity in RKO cells. Representative images were presented. Scale bar, 100 μm. **k-l** Transwell assay was performed to determine the migration ability of RKO cells. Representative images were presented. Scale bar, 100 μm. **m–o** HCT116 cells were subcutaneously injected into the nude mice (*n* = 5 per group). The tumor growth curve (**m**), photographs (**n**) and weight (**o**) were represented. The date were presented as means ± SD at least three independent experiments. Student’s *t* -tests (**d-f**, **i-l** and **o**) and two-way ANOVA test (**g**, **h** and **m**) were performed, ns: no significance, *p < 0.05, **p < 0.01, ***p < 0.001, ****p < 0.0001

To investigate MKK3's role in CRC, we established HCT116 and RKO cell lines overexpressing MKK3 (Fig. 5a) and MKK3 knocked out (Fig. 5b). Notably, the constitutively active MKK3-EE mutant markedly enhanced phosphorylation of its downstream target p38, while the inactive MKK3-AA mutant showed no such activation (Fig. 5c). Colony formation assays demonstrated that MKK3 overexpression significantly increased proliferative capacity in HCT116 cells (Fig. 5d). Furthermore, comparative analysis revealed that MKK3-EE exhibited stronger pro-proliferative effects compared to MKK3-AA (Fig. 5e), whereas MKK3 knockout substantially attenuated colony-forming ability (Fig. 5f). CCK-8 assays showed accelerated growth kinetics in MKK3-overexpressing cells, conversely, MKK3 knockout led to growth retardation (Fig. 5g, h). Consistently, EdU incorporation assay confirmed enhanced DNA synthesis in MKK3-overexpressing cells, whereas knockout cells displayed reduced synthesis activity (Fig. 5i, j). Transwell assay further validated MKK3's ability to promote cellular motility (Fig. 5k-l). In vivo xenograft model demonstrated that MKK3 significantly promoted tumor growth, as evidenced by the larger volume and heavier weight of the resulting tumors (Fig. 5m-o). Collectively, our results demonstrated MKK3 significantly enhances the proliferative, migratory, and clonogenic capacities of CRC cells.

### Blocking K32 ubiquitination stabilizes MKK3 and promotes colorectal cancer

**Fig. 6:**
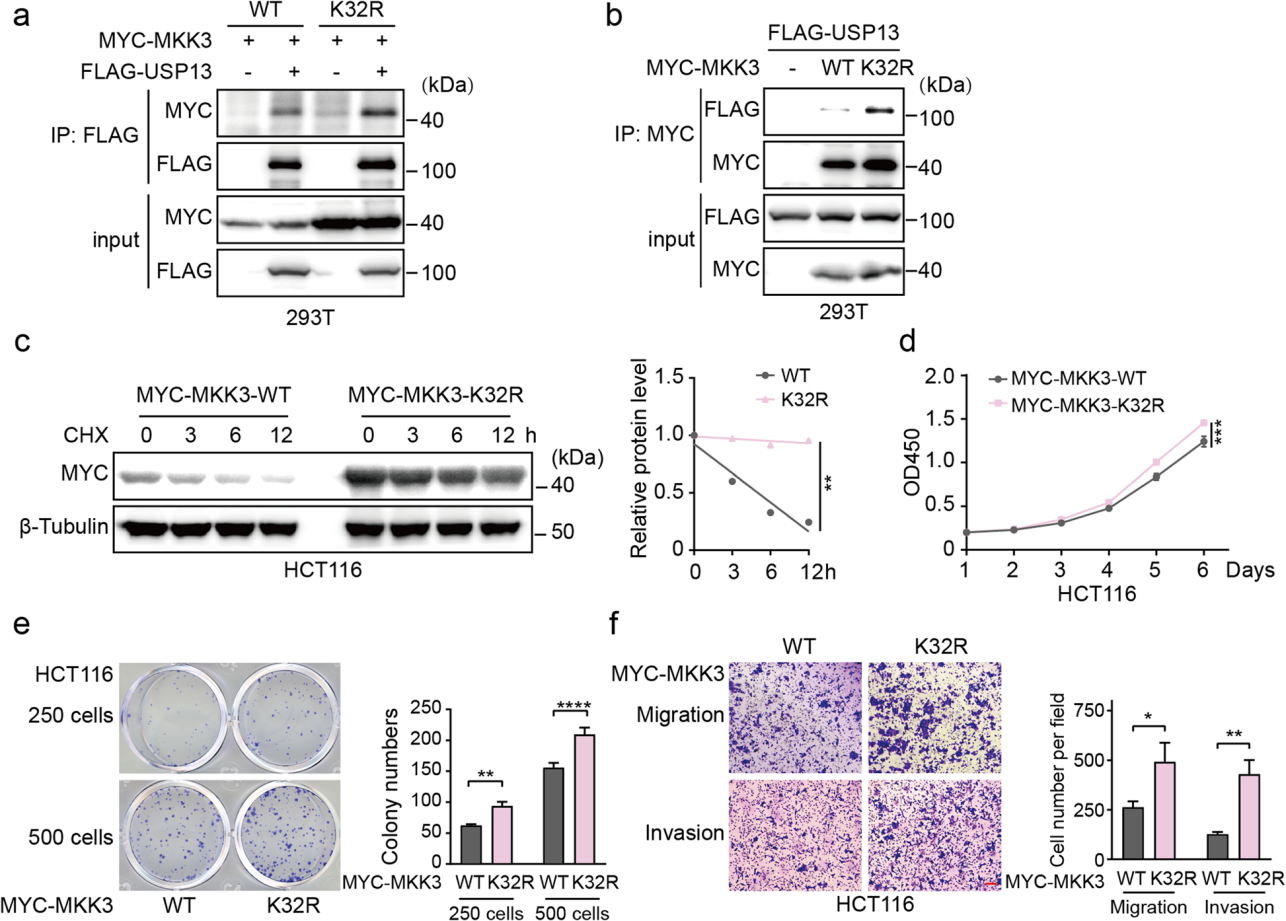
Blocking K32 ubiquitination stabilizes MKK3 and promotes colorectal cancer. **a-b** Lysates of HEK293T cells transfected with MYC-MKK3-WT or K32R and FLAG-USP13 as indicated were immunoprecipitated with anti-FLAG/MYC antibodies, followed by immunoblot analysis with the indicated antibodies. **c** HCT116 cells expressing MYC-MKK3-WT or K32R were treated with CHX (50 μg/mL), and cells were collected at indicated time points. The expression levels of exogenous MYC-MKK3 proteins were detect by Western blot. The gray values of the protein at each time point were quantified using ImageJ, and relative values were calculated by comparing with the loading control. A line graph was plotted to show the changes in protein levels over time. **d** CCK-8 assay was performed to detect cell proliferation ability of MYC-MKK3-WT or K32R overexpressing HCT116 cells. **e** Colony formation ability was determined in MYC-MKK3-WT or K32R overexpressing HCT116 cells. **f** Transwell assay was performed to determine the migration and invasion ability of MYC-MKK3-WT or K32R overexpressing HCT116 cells. Representative images were presented. Scale bar, 100 μm. The date were presented as means ± SD at least three independent experiments. Student’s *t* -tests (**e–f**) and two-way ANOVA test (**c-d**) were performed, *p < 0.05, **p < 0.01, ***p < 0.001, ****p < 0.0001

Building on the identification of K32 of MKK3 as the major USP13 deubiquitination site, we discovered that the K32R mutant exhibits enhanced USP13 binding and profound stabilization of in CHX-chase assays (Fig. 6a-c). Consistent with its enhanced protein stability, the K32R mutation significantly augmented the proliferative, clonogenic, and migratory capacities of HCT116 cells (Fig. 6d-f). In conclusion, the MKK3-K32R mutant significantly enhances the growth, proliferation, migration, and invasion capabilities of CRC cells.

These findings underscore the critical importance of the K32 site for MKK3 stability and its oncogenic function. We concluded that USP13 promotes CRC malignancy by specifically deubiquitinating K32 to stabilize MKK3.

### MKK3 alleviate USP13 deficient induced phenotype

**Fig. 7:**
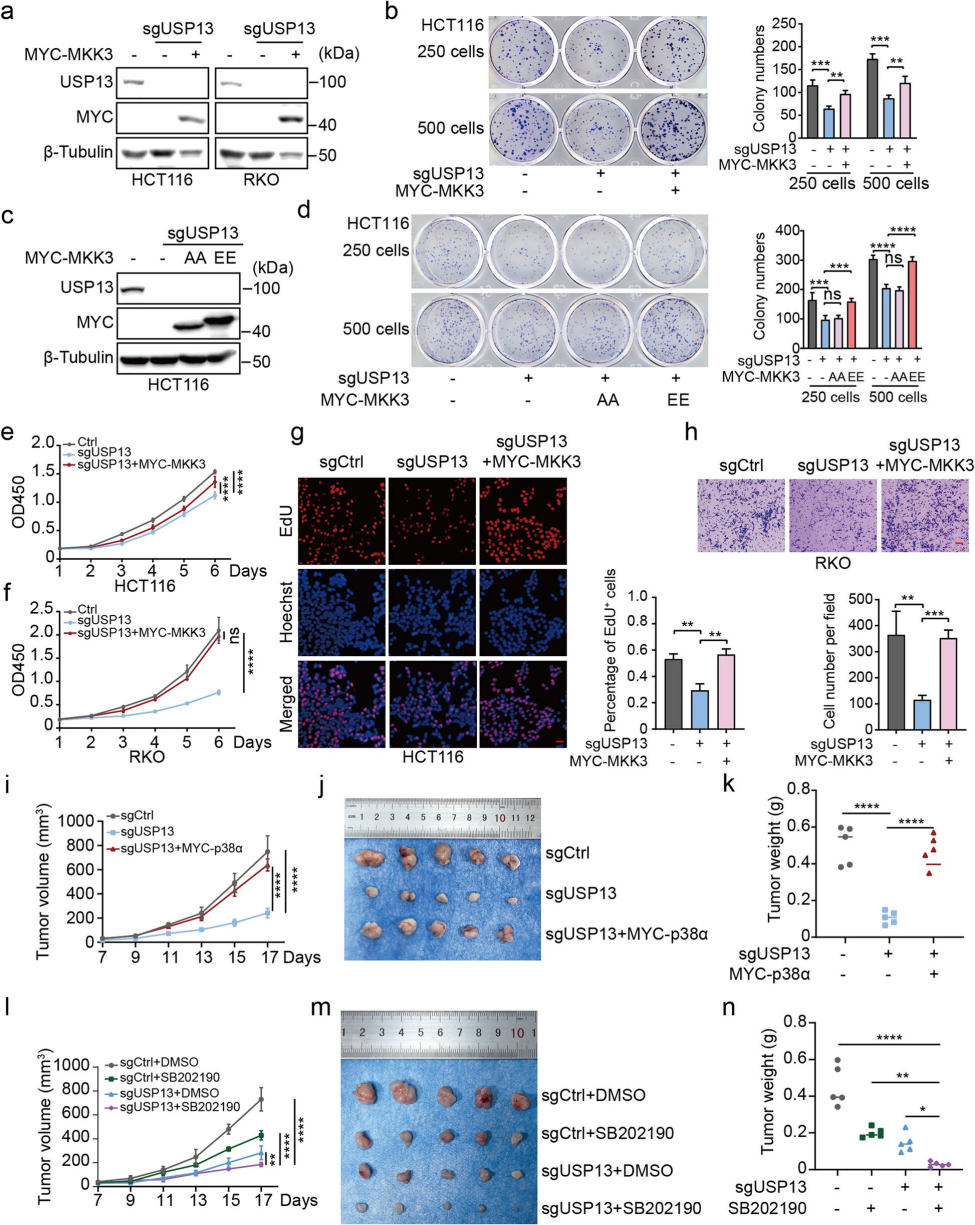
MKK3 alleviate USP13 deficient induced phenotype. **a** MYC-MKK3 was introduced into USP13-knockout cells. Western blot was performed to confirm USP13 knockout and exogenous MYC-MKK3 expression. **b** HCT116 cells were seeded into 12-well plates at a density of 250 or 500 cells per well. After 7 days, colonies were stained with crystal violet and quantified. **c** MYC-MKK3-AA/EE mutants were introduced into USP13-knockout cells. Western blot was performed to confirm USP13 knockout and exogenous MYC-MKK3 expression. **d** HCT116 cells were seeded into 12-well plates at a density of 250 or 500 cells per well. After 7 days, colonies were stained with crystal violet and quantified. **e–f** CCK-8 assay was performed to assess cell proliferation ability of USP13 knockout or MKK3 reconstitution HCT116 or RKO cells. **g** EdU assay was performed to assess the DNA synthesis activity in USP13 knockout or MKK3 reconstitution HCT116 cells. Representative images were presented. Scale bar, 100 μm. **h** Transwell assay was performed to assess the migration ability of USP13 knockout or MKK3 reconstitution RKO cells. Representative images were presented. Scale bar, 100 μm. **i-k** USP13 knockout or p38α reconstitution HCT116 cells were subcutaneously injected into the nude mice (*n* = 5 per group). **l-n** Control or USP13 knockout HCT116 cells were subcutaneously injected into the nude mice (*n* = 5 per group). Tumor-bearing mice were treated daily with either vehicle (10% DMSO, 40% PEG300, 5% Tween-80, and 45% saline, i.p.) or SB202190 (5 mg/kg in 10% DMSO, 40% PEG300, 5% Tween-80, and 45% saline, i.p.). The tumor growth curve (**i**, **l**), photographs (**j**, **m**) and weight (**k**, **n**) were represented. The date were presented as means ± SD at least three independent experiments. Student’s *t* -tests (**b**, **d**, **g**, **h**, **k** and **n**) and two-way ANOVA test (**e**, **f**, **i** and **l**) were performed, ns: no significance, *p < 0.05, **p < 0.01, ***p < 0.001, ****p < 0.0001

To determine whether USP13 promotes CRC progression through MKK3, we reintroduced MYC-MKK3 into USP13-knockout cell lines (Fig. 7a). Reintroduction of MKK3 rescued the impaired clonogenic capacity of USP13-knockout cells (Fig. 7b). Notably, the impaired clonogenicity was rescued by the phospho-mimetic MKK3-EE, but not by the kinase-dead MKK3-AA (Fig. 7c, d). In CCK-8 assays, reintroduction of MKK3 rescued the impaired growth rate of USP13-knockout CRC cells (Fig. 7e, f). Furthermore, the defect in DNA synthesis caused by USP13 knockout was reversed upon MKK3 reintroduction (Fig. 7g). Likewise, MKK3 reconstitution restored the cell migration defect observed in USP13-deficient cells (Fig. 7h). In summary, the reintroduction of MKK3 effectively rescued the reduced growth rate, decreased proliferative capacity, and impaired migratory ability caused by USP13 knockout. We conclude that USP13 promotes the onset and progression of CRC by stabilizing MKK3.

Finally, we demonstrated that p38 activation is both sufficient and necessary for the tumor-promoting effects downstream of the USP13-MKK3 axis. In both colony formation assays and xenograft models, the impaired tumor growth caused by USP13 knockout was effectively rescued by MYC-p38α overexpression (Fig. S3a-b; Fig. 7i-k). Pharmacological inhibition of p38 by SB202190 significantly attenuated tumor growth. Furthermore, this suppressive effect was profoundly enhanced by the additional knockout of USP13, leading to the greatest reduction in tumor size (Fig. 7l-n). Collectively, our in vivo results firmly establish the USP13-MKK3-p38 axis as a critical driver of colorectal tumorigenesis.

### USP13-MKK3-p38 promotes tumor formation

**Fig. 8:**
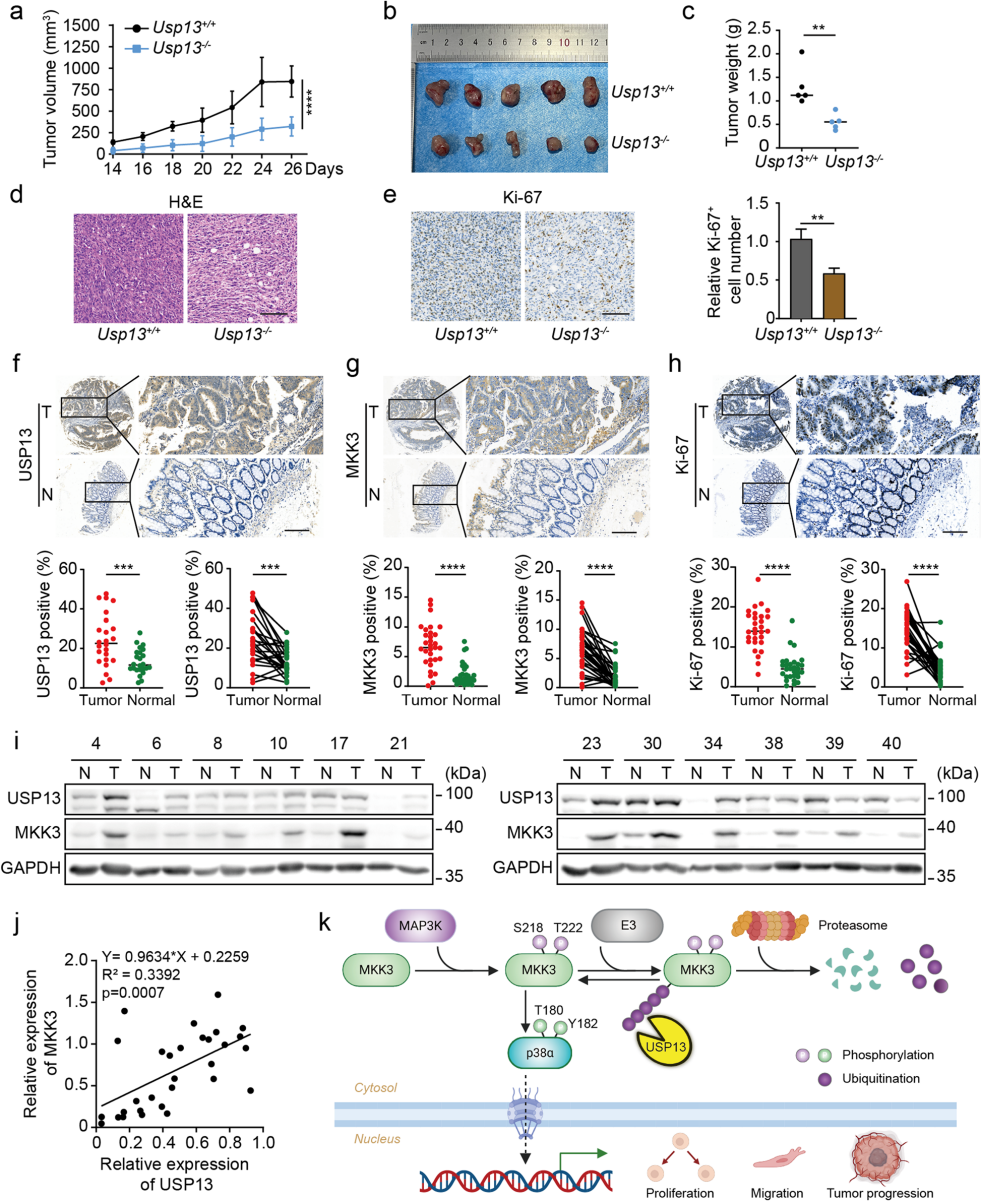
USP13-MKK3-p38 promotes tumor formation. **a-c** MC38 cells were subcutaneously injected into 8-week-old *Usp13*^+*/*+^ and *Usp13*^*−/−*^ C57 mice (*n* = 5 per group). Tumor size was measured every other day once tumors became visible. At the end of the experiment, tumors were excised, photographed, and weighed. The tumor growth curve (**a**), photographs (**b**) and weight (**c**) were represented. **d-e** H&E staining and Ki-67 immunostaining were performed on tumor samples, followed by quantification of the percentage of Ki-67-positive cells. Scale bar, 100 μm. **f–h** Tissue microarrays were prepared from CRC patient samples and stained with USP13, MKK3, and Ki-67 antibodies. The percentage of positive cells for each antibody was quantified and calculated (*n* = 30). Scale bar, 100 μm. **i** Western blot was performed to detect the protein levels of USP13 and MKK3 in CRC samples. The representative images were presented. **j **The band density of USP13 and MKK3 in was quantified using ImageJ software and normalized to the corresponding loading control. The correlation analysis of USP13 and MKK3 protein levels in 30 CRC samples. **k** Mechanism diagram of USP13-mediated stabilization of MKK3 through deubiquitination (Created by Biorender: https://BioRender.com/jze3xva). The date was presented as means ± SD at least three independent experiments. Student’s *t* -tests (**c** and **e–h**) and two-way ANOVA test (**a**) were performed, *p < 0.05, **p < 0.01, ***p < 0.001, ****p < 0.0001

We implanted murine CRC cell MC38 into USP13 knockout (*Usp13*^*−/−*^) mice to evaluate the impact of USP13 on CRC progression in vivo. As shown in Fig. 8a-c, tumors from *Usp13*^*−/−*^ mice exhibited significantly impaired growth, as assessed by reduced growth rate, tumor volume, and weight compared to wild-type controls. The Ki-67 expression in tumors derived from *Usp13*^*−/−*^ mice was also reduced, indicating lower proliferative activity (Fig. 8d-e). This indicated that USP13 fosters a tumor-supportive microenvironment in vivo, and its knockout consequently compromises tumor cell colonization and proliferation.

After observing the tumor-promoting effect of USP13 on CRC development in the mouse model, we further examined the expression of USP13, MKK3, and Ki-67 in CRC tissues from patients using immunohistochemistry (IHC). As shown in Fig. 8f-h, a comparative analysis of tissue microarrays from CRC patients revealed that USP13 and MKK3 were significantly overexpressed in tumor tissues compared to adjacent normal tissues. Additionally, Ki-67 expression was also markedly higher in CRC tissues than in adjacent tissues. Statistical analysis confirmed that, regardless of whether the samples were paired or unpaired, the expression levels of USP13, MKK3, and Ki-67 were consistently and significantly elevated in tumor tissues compared to adjacent normal tissues. We further collected tissue samples from 30 CRC patients and analyzed the protein levels of USP13 and MKK3. The results showed that USP13 and MKK3 expression was significantly elevated in tumor tissues compared to adjacent normal tissues (Fig. 8i). We then quantified the protein expression levels of USP13 and MKK3 using ImageJ and performed a correlation analysis. As shown in the Fig. 8j, USP13 and MKK3 exhibited a positive correlation, suggesting a potential link between their expression in CRC progression.

## Discussion

The activity of MAPK pathway proteins is primarily regulated by phosphorylation, while protein levels are controlled by ubiquitination. Research on the ubiquitination of MAPK pathway proteins has mainly focused on MAP3Ks, such as RAF, TAK1, and ASK1. RAF proteins, particularly the BRAF^V600E^ mutation, are frequently found in various cancers and are commonly targeted in drug therapies [42]. TAK1, in addition to participating in the MAPK pathway signal cascade, also plays a role in the NF-κB signaling pathway, regulating inflammation and innate immunity. Ubiquitination not only affects the stability of TAK1 but also influences its interactions with other proteins and its kinase activity [43–45]. ASK1 is activated by ROS, hydrogen peroxide, and TNF, and induces cell death and inflammation through the activation of p38 and JNK. The protein levels of ASK1 are regulated by ubiquitination [46, 47]. However, the ubiquitination of other MAPK pathway proteins remains poorly characterized, such as JNK, p38, and their direct upstream kinases (MAP2Ks like MKK3, MKK4, MKK6, and MKK7). Only a few studies have explored their ubiquitination, making the investigation of their ubiquitination and deubiquitination modifications an area that requires further research [20, 21, 48].

This study systematically reveals the molecular mechanism by which the deubiquitinase USP13 promotes the malignant progression of CRC by targeting MKK3, regulating the p38 phosphorylation cascade. Specifically, USP13 directly interacts with the C-terminal domain of MKK3 through its UBA domain and, relying on its deubiquitinase activity, selectively removes K48-linked ubiquitination (especially at the K32 site) on MKK3, thereby inhibiting its degradation via the proteasome. The stabilized MKK3 continuously activates the downstream p38 signaling pathway by enhancing its phosphorylation levels (Ser218/Thr222), driving CRC proliferation, migration, and in vivo tumorigenic capacity (Fig. 8k). Importantly, this regulatory process is subject to positive feedback regulation by MKK3 phosphorylation status: although the constitutively active MKK3-EE mutant degrades more rapidly, it binds more strongly to USP13, and USP13 has a more significant stabilizing effect on MKK3-EE, forming an "activation-deubiquitination-reactivation" amplification loop. Meanwhile, blocking ubiquitination at the K32 site of MKK3 (K32R mutation) mimics the USP13 overexpression phenotype, further confirming that this site is the core target of the USP13-MKK3 regulatory axis. However, the specific in vivo function of this site requires further investigation through cellular in situ point mutation and corresponding mouse model.

The phosphorylation-dependent degradation mechanism of MKK3 has not been previously reported. This study is the first to reveal a dynamic correlation between MKK3 activation (Ser218/Thr222 phosphorylation) and its ubiquitination. The ubiquitination level of activated MKK3-EE is significantly higher than that of inactivated MKK3-AA, possibly because MKK3 phosphorylation enhances its interaction with potential E3 ubiquitin ligases. Phosphorylation may induce structural changes that facilitate ubiquitination. For example, site-specific phosphorylation expose recognition sites for ubiquitin ligases or alter protein conformation, making them more accessible to ubiquitination machinery [49, 50]. Further studies involving mass spectrometry and protein interaction assays are required to identify potential E3 ligases and additional ubiquitination sites associated with this process.

MKK3 plays a pro-tumorigenic role in various types of cancer [12]. By activating the p38 MAPK signaling pathway, MKK3 influences key cellular processes such as cell cycle progression, proliferation, and apoptosis. For instance, overexpression of MKK3 leads to elevated p38 phosphorylation, thereby promoting cancer cell proliferation. Conversely, MKK3 knockout or inhibition significantly suppresses cancer cell growth [16]. Beyond proliferation, MKK3 is also crucial for cancer cell migration and invasion [51]. Our study has shown that MKK3 overexpression enhances the migratory capacity of CRC, whereas its knockout inhibits migration. MKK3 facilitates cytoskeletal remodeling by activating downstream effectors of the p38 MAPK pathway, thereby promoting cell motility and invasiveness. Additionally, MKK3 exerts anti-apoptotic effects, particularly under environmental stress conditions such as oxidative stress or cellular damage. By activating the p38/MAPK pathway, MKK3 enables cancer cells to survive under adverse conditions, thereby increasing their resistance to therapy and immune surveillance [52]. This survival advantage accelerates tumor progression, making MKK3 a potential therapeutic target for cancer treatment.

Our understanding of the USP13-MKK3 axis in this study primarily focuses on CRC, while its role in other cancers remains insufficiently explored. Given the diverse genetic mutation backgrounds of different cancers, the function of MKK3 may vary across cancer types. Therefore, further investigation is needed to elucidate the specific roles and mechanisms of the USP13-MKK3 axis in different cancers. MKK3 is involved in multiple physiological processes, including stress response and immune regulation [53]. Inhibiting MKK3 could have unpredictable effects on normal cellular functions, such as impairing the cellular response to oxidative stress and increasing the risk of cellular damage. Additionally, whether the USP13-MKK3 axis can be effectively combined with existing targeted therapies (such as MEK, PI3K, and BRAF inhibitors) or immunotherapies (such as PD-1/PD-L1 antibodies) to enhance therapeutic efficacy requires further clinical trial data. Furthermore, the potential of USP13 or MKK3 as prognostic or predictive biomarkers for cancer treatment response necessitates additional validation using clinical samples and bioinformatics analyses.

In conclusion, our study identifies the deubiquitinase USP13 as a critical regulator of the MKK3/p38 signaling axis in CRC. We elucidate a novel molecular mechanism whereby USP13 stabilizes MKK3 by removing its K48-linked ubiquitination, thereby enhancing p38 activation and driving tumor malignancy (Fig. 8k). Furthermore, the discovery of a positive feedback loop, linking MKK3 phosphorylation to its increased binding and stabilization by USP13, adds a new layer of complexity to the post-translational regulation of this pathway. These new findings highlight the potential of targeting the USP13-MKK3-p38/MAPK axis for CRC treatment.

## Materials and methods

### Cell culture and transfection

HEK293T, RKO, and SW48 cells were cultured in high-glucose DMEM, while HCT116 and HCT15 cells were maintained in McCoy's 5 A and RPMI-1640 medium, respectively. All media were supplemented with 10% fetal bovine serum (FBS) and 100 U/mL penicillin–streptomycin, and cells were incubated at 37 °C in a 5% CO₂ atmosphere. Transfections were performed using polyethylenimine (#24,765, Polysciences, USA) following the manufacturer's instructions.

### Plasmids and reagents

Plasmids expressing FLAG-USP13 and MYC-MKK3/MKK6 were generated by PCR and cloned into pHAGE-puro-6-tag. MYC-MKK3 mutants were generated by point mutation. HA-Ub and its mutants and FLAG-USP13-AE were kindly provided by professor Zhong Bo (Wuhan University, Wuhan, China). Anti-mouse β-Tubulin antibody (#AC021, 1:5,000) was purchased from Abclonal. Anti-rabbit antibody against MKK3 (#8535, 1:1,000), phospho-MKK3 (#12,289, 1:1,000), E-Cadherin (#3195, 1:2,000), Snail (#3879, 1:1,000), p38 (#8690, 1:2,000), phospho-p38 (#4511, 1:1,000), Erk1/2 (#4695, 1:2,000), phospho-Erk1/2 (#4370, 1:1,000), JNK (#67,096, 1:2,000) and phospho-JNK (#4668, 1:1,000) were purchased from CST and anti-mouse antibody against USP13 (#66,176–1-Ig, 1:2,000) were purchase from Proteintech. Anti-HA (#SLAB0202, 1:5,000), anti-MYC (#SLAB2903, 1:5,000) and anti-FLAG (#SLAB0102, 1:5,000) antibodies were purchased from Smart-Lifesciences (Changzhou Smart-Lifesciences Biotechnology, China). MG132 (#HY-13259), CHX (#HY-12320), Puromycin (#HY-B1743) and SB202190 (#HY-10295) were purchased from MCE (USA).

### Stable cell lines and knockout cell lines construction

Lentiviruses were produced by co-transfecting 293 T cells with the target plasmids with the packaging vectors pMD2.G (#12,259, Addgene) and psPAX2 (#12,260, Addgene). At 48 h post-transfection, viral supernatants were collected and used to infect CRC cells. Infected cells were selected with puromycin (5 mg/mL). For overexpression stable lines, pooled cells were maintained and validated via immunoblotting. For knockout lines, single-cell clones were isolated by limited dilution, expanded, and verified through both western blot analysis and sequencing.

### Western blot

Cells were harvested, washed with ice-cold PBS, and lysed in lysis buffer (50 mM Tris–HCl, pH 7.4, 150 mM NaCl, 1 mM EDTA, pH 8.0, 1% NP-40) supplemented with protease and phosphatase inhibitors. Protein concentrations were determined prior to loading. Equal amounts of protein samples were separated by SDS-PAGE and subsequently transferred onto polyvinylidene fluoride (PVDF) membranes (#IPVH00010, Millipore, Germany). The membranes were then blocked with 5% skim milk (#P0216, Beyotime, China) in TBST (20 mM Tris–HCl, pH 7.5, 150 mM NaCl, 0.1% Tween-20) for 1 h at room temperature, and then incubated with specific primary antibodies overnight at 4 °C. After washing, the membranes were probed with horseradish peroxidase (HRP)-conjugated secondary antibodies for 1 h at room temperature.. Protein bands were visualized using Clarity Western ECL Substrate (#1,705,061, Bio-Rad, USA) and detected with a ChemiDoc XRS + imaging system (#1,708,265, Bio-Rad, USA).

### Immunoprecipitation

Cells were harvested, washed with cold PBS, and lysed on ice with IP lysis buffer (50 mM Tris–HCl, pH 7.4, 150 mM NaCl, 1 mM EDTA, pH 8.0, 1% NP-40) containing protease and phosphatase inhibitors. Following centrifugation at 12,000 g for 15 min at 4 °C, the supernatants were incubated overnight at 4 °C with specific antibodies and rProtein A/G Beads (#SA032025, Smart-Lifesciences). The beads were subsequently washed three times with lysis buffer, and the immunoprecipitated proteins were eluted and analyzed by SDS-PAGE and immunoblotting.

### Ubiquitination assay

To detect protein ubiquitination, cells were first treated with the proteasome inhibitor MG132 (10 µM) for 12 h before harvesting to stabilize ubiquitinated proteins. Cells were then lysed using IP lysis buffer and denatured at 95 °C for 5 min in the presence of 0.1% SDS. The supernatants were incubated with the indicated antibodies and rProtein A/G Beads at 4 °C overnight. The beads were then washed three times with lysis buffer and protein ubiquitination was detected by Western blot.

### Immunofluorescence

Cells were seeded on 15-mm glass coverslips (#801,007, NEST, Wuxi, China) and allowed to adhere. After washing with PBS, cells were fixed with 4% paraformaldehyde (#P804537, Macklin, Shanghai, China) in PBS for 10 min at room temperature followed by three PBS washes. Cells were then permeabilized with 0.5% Triton X-100 (#30,188,928, Sinopharm, Shanghai, China) in PBS for 10 min and blocked with 3% BSA (#BD118650, Bidepharmatech, Shanghai, China) in PBS for 1 h at room temperature. Subsequently, the cells were incubated overnight at 4 °C with primary antibodies against MKK3 (1:200) and USP13 (1:200) diluted in 3% BSA/PBS. After three washes with PBS, the cells were incubated with Alexa Fluor 488-conjugated (#A11001, Invitrogen; 1:1000) and Alexa Fluor 594-conjugated (#21,207, Invitrogen; 1:1000) for 1 h at room temperature. Nuclei were counterstained with DAPI and images were acquired using an Olympus laser scanning confocal microscope.

### Cell proliferation

Cells were seeded into 96-well plates at 1,000 cells per well, with three replicates per condition. After 12 h, the medium was replaced with fresh medium containing CCK-8 reagent at a ratio of 1: 10 (CCK-8: DMEM). The plate was incubated at 37 °C with 5% CO₂ for 1 h. Absorbance at 450 nm was measured using a microplate reader. Cell proliferation was measured every 24 h to monitor growth over time.

### Colony formation

Cells were seeded into 6-well plates at 250/500 cells per well, with three replicates per condition. The plates were incubated at 37 °C in a 5% CO₂ atmosphere until visible colonies had formed. The culture medium was carefully aspirated, and the wells were gently washed with PBS. Cell fixation was performed using either 4% paraformaldehyde or methanol for 15 min at room temperature. The colonies were stained with 0.5% crystal violet solution for 10–30 min. The wells were thoroughly rinsed with distilled water to remove excess dye and colony numbers were counted.

### Cell migration and invasion

Cell migration and invasion assays were performed using Transwell chambers. For the invasion assay, the Transwell membrane was pre-coated with 50–100 μL of Matrigel and allowed to polymerize at 37 °C for 30–60 min prior to use, whereas for the migration assay, uncoated inserts were employed. Cells were harvested and resuspended in serum-free medium at a density of 100,000 cells in 200–300 μL. This cell suspension was carefully added to the upper chamber of each insert. The lower chamber was filled with complete medium supplemented with serum to serve as a chemoattractant. The plates were incubated at 37 °C in a 5% CO₂ atmosphere for 12–24 h (migration) or 24–48 h (invasion). Following incubation, non-migratory/non-invasive cells on the upper surface of the membrane were removed gently with a cotton swab. Cells that had penetrated to the lower surface were washed with PBS, fixed with 4% paraformaldehyde or methanol for 15 min, and stained with 0.5% crystal violet for 15–30 min. After rinsing with distilled water, images of the stained cells were captured using a light microscope. The number of migrated or invaded cells was quantified by counting at least five randomly selected fields per membrane.

### Immunohistochemistry

The human CRC tissue samples used in this study were obtained from the Second Affiliated Hospital, University of South China (approval No.2024085). Surgical tumor specimens from CRC patients and xenograft-derived tumors were preserved in 4% paraformaldehyde, embedded in paraffin, and sliced into 4 μm thick sections. These sections underwent deparaffinization, rehydration, and treatment with 3% hydrogen peroxide for 10 min to inhibit endogenous peroxidase activity. The slides were then incubated overnight at 4 °C with primary antibodies targeting USP13 (Proteintech, 1:200), MKK3 (CST, 1:200), and Ki-67 (CST, 1:200), after a 1-h blocking step using 5% BSA at room temperature. Subsequently, the slides were treated with HRP-conjugated secondary antibodies for 1 h at room temperature. For chromogenic visualization, DAB substrate was applied and incubated for 1–5 min until brown staining appeared. Finally, the slides were mounted and analyzed under a light microscope for DAB staining.

### Xenograft tumor experiment

Tumor cells were cultured to the logarithmic growth phase and harvested with trypsin. After collection, the cells were resuspended in PBS at a density of 1 × 10⁶ to 1 × 10⁷ cells/mL. This cell suspension was thoroughly mixed with Matrigel at a 1:1 ratio on ice. Mice were randomly assigned to experimental and control groups (n ≥ 5) and subsequently inoculated subcutaneously in the flank or dorsal region with 100–200 μL of the cell-Matrigel mixture under sterile conditions. Animal health status was assessed daily. Beginning on day 3 post-inoculation, tumor length (*L*) and width (*W*) were measured every 2–3 days using a digital caliper. Tumor volume (*V*) was calculated according to the formula: *V* = 0.5 × *L* × *W*^*2*^. When the tumor volume reached 100 mm^3^, tumor-bearing mice were treated daily with either vehicle (10% DMSO, 40% PEG300, 5% Tween-80, and 45% saline, i.p.) or SB202190 (5 mg/kg in 10% DMSO, 40% PEG300, 5% Tween-80, and 45% saline, i.p.). When the average tumor volume reached 1000–1500 mm3, or when mice exhibited significant weight loss or restricted mobility, the animals were euthanized, and subcutaneous tumors were surgically dissected.

### Statistical analysis

All data were analyzed using GraphPad Prism 9.0. Quantitative data from at least three independent biological replicates are presented as the mean ± standard deviation. Statistical significance between two groups was determined using a two-tailed Student's *t*-test. For comparisons among more than two groups, One-way or Two-way ANOVA was performed, as appropriate. Correlations were assessed using Spearman's correlation analysis. A P-value of less than 0.05 was considered statistically significant.

## Supplementary Information

Supplementary Material 1.

## Data Availability

All relevant data are within the paper. All materials are available upon reasonable request from the corresponding authors.
