# Supplementary material for: Ubiquitin-specific protease 13 promotes colorectal cancer progression by stabilizing mitogen-activated protein kinase kinase 3
Source: Mol Biomed. 2025 Nov 27;6:122. doi: 10.1186/s43556-025-00375-3 (PMC12660564; doi:10.1186/s43556-025-00375-3)
Supplement: Supplementary file 1 — Supplementary Material 1. [file 43556_2025_375_MOESM1_ESM.docx]

**Supplementary Information**

**Ubiquitin-specific protease 13 promotes colorectal cancer progression by stabilizing mitogen-activated protein kinase kinase 3**

Si-yu Chang^1†^, Bo Gu^2†^, Yong Xiong^1^, Meng-si Zhao^1^, Le Li^1^, Yi Liu^1^, Bai-qi Wang^1^, Guo-qing Li^1^, Run-lei Du^1, 2*^, Xiao-dong Zhang^1, 2*^

^1^National Health Commission Key Laboratory of Birth Defect Research and Prevention, MOE Key Lab of Rare Pediatric Diseases, Hunan Provincial Key Laboratory of Basic and Clinical Pharmacological Research of Gastrointestinal Cancer, the Second Affiliated Hospital, School of Basic Medical Sciences, Hengyang Medical School, University of South China, Hengyang, China.

^2^Hubei Key Laboratory of Cell Homeostasis, College of Life Sciences, Wuhan University, Wuhan, 430072, Hubei China.

^*^Correspondence:

Run-lei Du: runleidu@whu.edu.cn, Xiao-dong Zhang: zhangxd@usc.edu.cn

^†^These authors contributed equally to this work.

Supplemental Figure 1


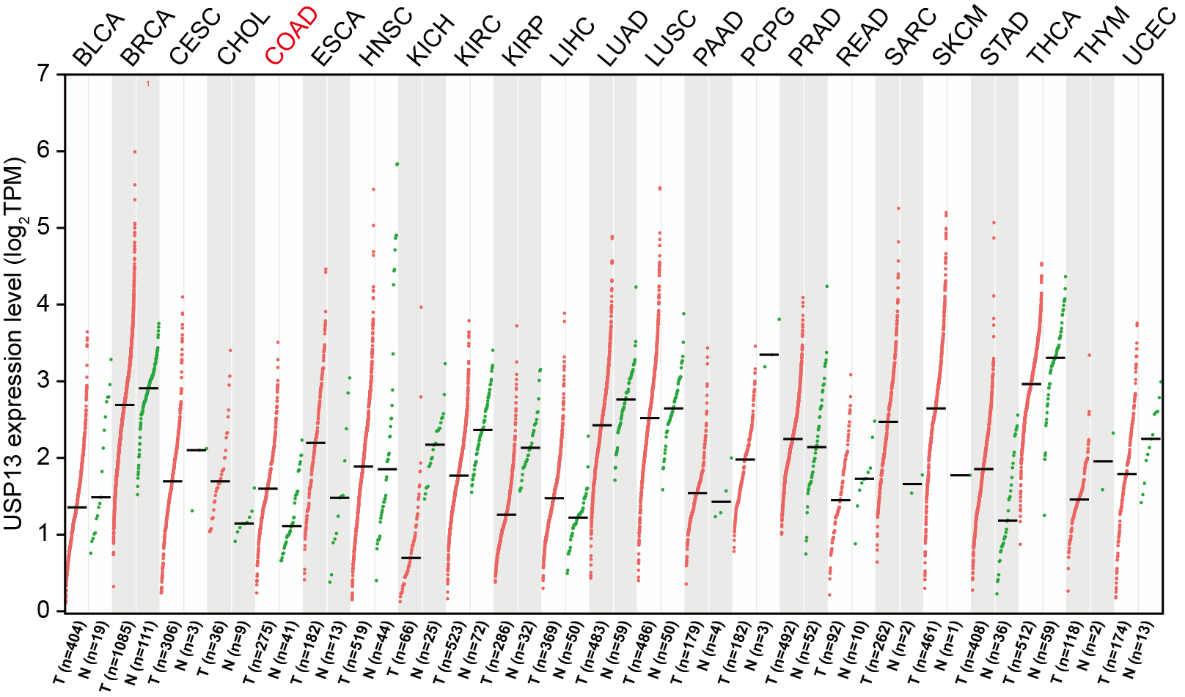


**Fig. S1 USP13 is upregulated in COAD.** The expression levels of USP13 in tumor and normal tissues across different types of cancer were analyzed using the GEPIA2 (Gene Expression Profiling Interactive Analysis) database (http://gepia2.cancer-pku.cn).

Supplemental Figure 2


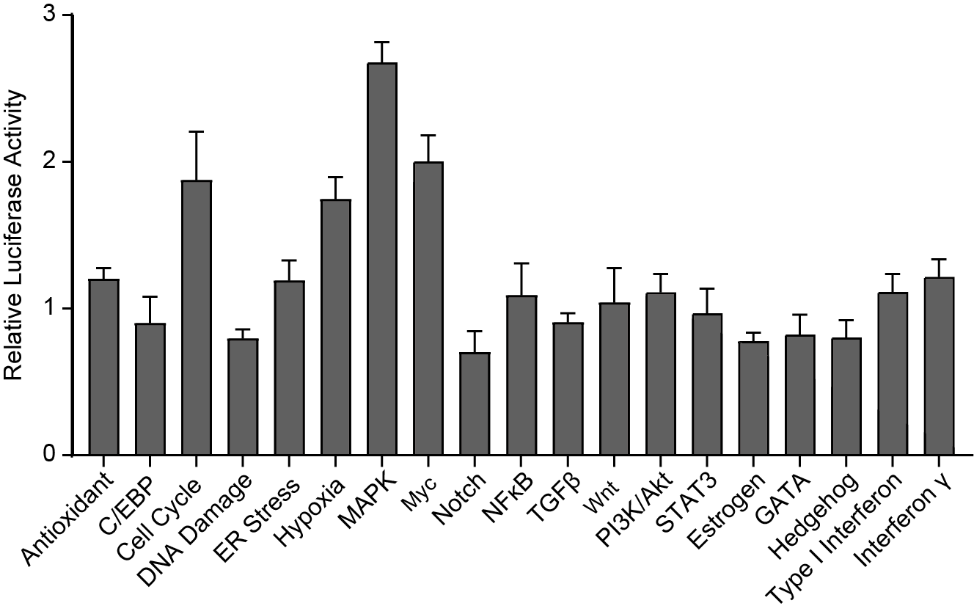


**Fig. S2 Identification of USP13-affected cell signaling pathways using luciferase reporter assays.** HEK293T cells were co-transfected with a pGL3-TK-luc reporter plasmid and a series of pathway-specific response element constructs. Firefly luciferase activity was measured and normalized to Renilla. The graph represents the relative change in luciferase activity induced by USP13 overexpression compared to the empty vector control.

Supplemental Figure 3


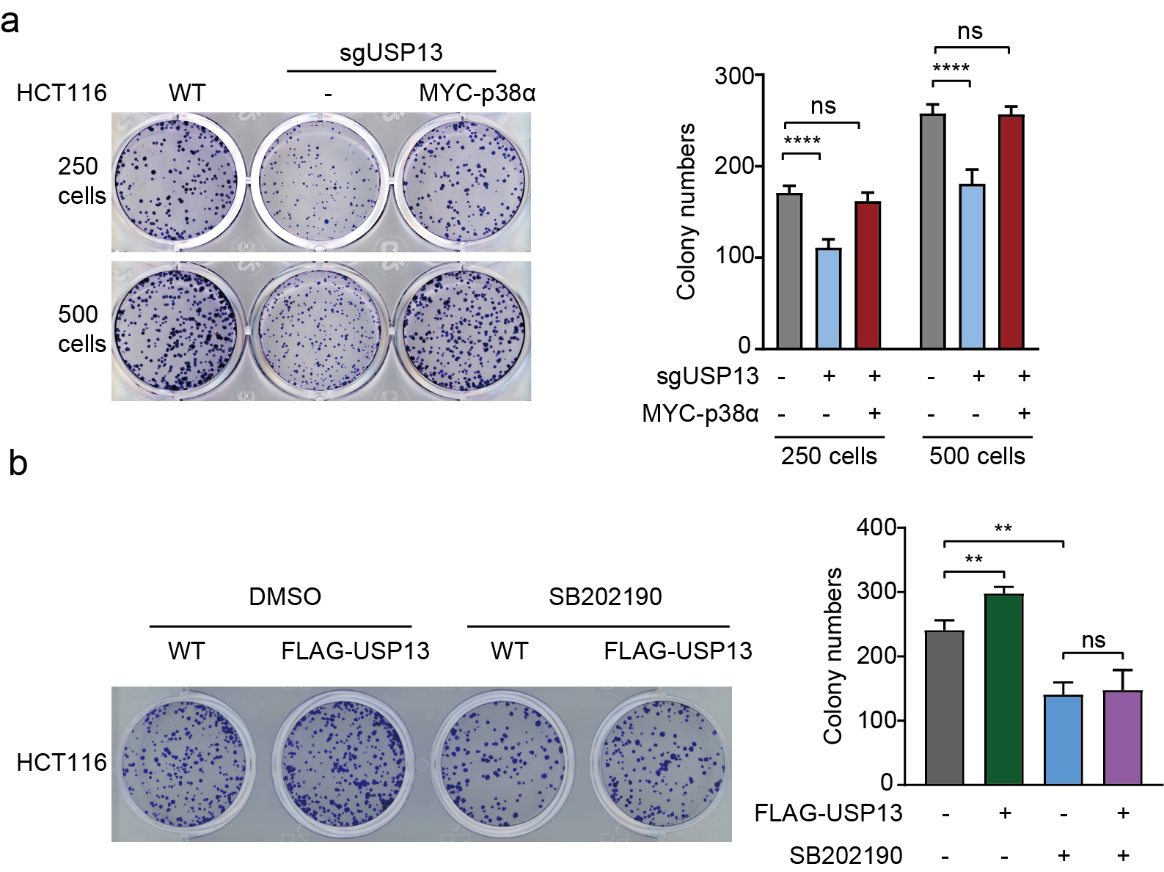


**Fig. S3 USP13-mediated promotion of CRC cell proliferation is dependent on the p38 pathway.** **a.** Colony formation ability was assessed in USP13 knockout or p38 reconstitution HCT116 cells. **b.** Colony formation ability was assessed in control or USP13 overexpressing HCT116 cells were treated with p38 inhibitor SB202190 (20 μM).

Student’s *t* -tests were performed with mean ± SD, ns: no significance, **p < 0.01, ****p < 0.0001.
